# GUTAID: a curated database linking gut microbial antigens to autoimmune mechanisms

**DOI:** 10.1093/database/baag029

**Published:** 2026-06-03

**Authors:** Laibah Hashmi, Shoaib Ur Rehman, Farzana Jabeen, Masood Ur Rehman Kayani

**Affiliations:** Metagenomics Discovery Lab, Department of Sciences, School of Interdisciplinary Engineering & Sciences (SINES), National University of Sciences & Technology (NUST), Srinagar Highway, Sector H-12, Islamabad 44000, Pakistan; Metagenomics Discovery Lab, Department of Sciences, School of Interdisciplinary Engineering & Sciences (SINES), National University of Sciences & Technology (NUST), Srinagar Highway, Sector H-12, Islamabad 44000, Pakistan; School of Electrical Engineering and Computer Science (SEECS), National University of Sciences & Technology (NUST), Srinagar Highway, Sector H-12, Islamabad 44000, Pakistan; Metagenomics Discovery Lab, Department of Sciences, School of Interdisciplinary Engineering & Sciences (SINES), National University of Sciences & Technology (NUST), Srinagar Highway, Sector H-12, Islamabad 44000, Pakistan

## Abstract

Gut dysbiosis is widely recognized as a contributor to autoimmune diseases, as it can lead to the expression of microbial antigens that disrupt immune regulation through specific molecular mechanisms. However, existing resources do not systematically link gut microbial antigen sequences to the specific autoimmune mechanisms through which they act. Here, we present GUTAID (Gut Microbes in Autoimmune Disorders), a literature-curated database of gut microbial antigens annotated with experimentally supported autoimmune mechanisms. Peer-reviewed studies published from October 1970 to September 2024 were manually screened, yielding 73 potential antigens that operate through nine molecular mechanisms, including protein citrullination, epitope spreading, molecular mimicry, and immune modulation, amongst others. The corresponding protein sequences were retrieved from UniProtKB, and redundancy was removed with MMseqs2. For the database implementation, data were delivered through a lightweight LAMP (Linux–Apache–MySQL/MariaDB–PHP) stack with server-side HTML/Bootstrap rendering, MySQL indexing, and HTTPS-secured downloads. Users can browse, keyword-search, or bulk-download sequence archives via a five-tab interface (Home, Downloads, Search, Team, and About). GUTAID thus enables mechanism-oriented exploration of gut microbial antigens and supports downstream biomarker and therapeutic discovery in autoimmune research. **Database URL:**  https://gutaid.mgdiscoverylab.com/

## Introduction

Autoimmune diseases are chronic disorders in which the immune system starts attacking the body’s own tissues, leading to persistent inflammation and organ damage. Collectively, these conditions affect approximately 3%–8% of the global population and have a higher prevalence in females. They also impose a substantial clinical and economic burden [1]. Clinical manifestations may involve a single organ, as in autoimmune hepatitis or vitiligo, or present as systemic diseases such as multiple sclerosis and type 1 diabetes. This diversity highlights the wide range of tissues that may be targeted [2]. In recent years, growing evidence has identified disruptions in the gut microbiome as a common environmental factor capable of disturbing immune homeostasis and contributing to the development of autoimmunity [3].

Recent studies have shown that gut–microbiome dysbiosis is a pathogenic cofactor in rheumatoid arthritis (RA). These studies report consistent shifts in microbial community structure and reduced taxonomic diversity in patients compared with healthy controls [4–6]. Mechanistically, dysbiosis promotes autoimmunity in several ways. First, impaired intestinal barrier function allows luminal antigens to enter the bloodstream, allowing access to mucosal immune cells and triggering systemic autoreactivity [7, 8]. Second, increased antigenic overlap between microbial and host proteins leads to molecular mimicry and loss of immune tolerance [9, 10]. Third, an enrichment of microbes and viruses that amplify pro-inflammatory cytokine networks linked to cartilage and bone destruction [11, 12]. Together, these studies indicate that the gut microbiome acts as a modulatory layer that influences autoimmune risk factors, thereby influencing the onset and progression of RA and related autoimmune disorders.

It is also important to note how these altered microbial communities translate into immune dysfunction. In the literature curated for GUTAID (Gut Microbes in Autoimmune Disorders), gut microbial antigens were linked to nine recurring autoimmune mechanism categories. These include molecular mimicry, superantigen activation, immune activation, epitope spreading, chronic infection, protein citrullination, immune modulation, inflammation-induced response, and immune system disruption. Molecular mimicry arises when microbial epitopes resemble host proteins, activating cross-reactive T- and B-cell clones that mistake self-antigens as targets. Persistent inflammation facilitates epitope spreading, in which newly exposed self-determinants become secondary immune targets [13, 14]. Superantigen activation can bypass normal peptide specificity by crosslinking MHC class II molecules with T-cell receptors, resulting in broad T-cell activation and abnormal helper signals that may promote autoreactivity [15, 16]. More broadly, sustained immune activation, chronic infection, and inflammation-induced responses can maintain prolonged antigen exposure, cytokine release, and tissue damage. This lowers the threshold for autoreactive lymphocyte activation and perpetuates autoimmune pathology [17–19]. Protein citrullination contributes by generating neoepitopes that can be recognized as non-self. Altered immune modulation or immune system disruption can weaken regulatory pathways that normally preserve immune homeostasis and self-tolerance [20–22]. Understanding how these microbial antigens exploit these mechanisms could guide biomarker discovery and inspire microbiome-targeted interventions for autoimmune diseases. This would pave the way for enhanced disease management.

To achieve this goal, researchers need a unified resource that connects gut antigens with their specific modes of immune disruption. Existing databases only partially meet this need. The Immune Epitope Database (IEDB) compiles pathogen, allergen, and self-epitopes but rarely specifies the microbial source or the mechanism underlying autoimmune involvement [23]. Microbe disease catalogues such as HMDAD and gutMDisorder provide taxon-level associations without sequence-level data [24, 25], whereas vaccine-oriented repositories (e.g. VIOLIN) prioritize protective antigens rather than those that break tolerance [26]. Prediction tools like VaxiJen offer *in silico* antigenicity scores with no experimental confirmation or mechanistic annotation [27]. Consequently, information about microbial antigens that act through molecular mimicry, epitope spreading, or bystander activation remains scattered across individual studies. GUTAID addresses this gap by consolidating experimentally verified gut antigens and explicitly tagging each sequence with its reported autoimmune mechanism. It thereby provides a single, curated platform for mechanism-oriented research.

GUTAID is the first literature-curated database dedicated to gut microbial antigenic sequences associated with well-defined autoimmune mechanisms. IEDB, HMDAD, and gutMDisorder focus on epitopes or taxon–disease associations rather than per-antigen mechanistic mapping. GUTAID complements these resources by explicitly connecting each curated antigen to a defined autoimmune mechanism. By integrating mechanistic annotation with sequence-level data, GUTAID offers three practical advantages. (i) It eliminates the laborious task of manually screening scattered literature sources to collect relevant information. (ii) It supplies a set of non-redundant, sequence-verified antigens that are ready for downstream bioinformatics and experimental workflows. (iii) It allows one-click FASTA downloads for immediate use without any need for editing. Collectively, these capabilities establish GUTAID as a foundational reference for aiding mechanistic research on autoimmune disorders.

## Materials and methods

### Data collection

Peer-reviewed studies published from October 1970 to September 2024 were searched in PubMed and Google Scholar with the keywords gut microbiome, intestinal microbiota, autoimmune disease (including disease-specific names), and autoimmune mechanisms (molecular mimicry, superantigen activation, immune activation, epitope spreading, chronic infection, protein citrullination, immune modulation, inflammation-induced response, immune system disruption). The search strategy prioritized studies describing autoimmune disease mechanisms in human subjects; however, studies employing validated animal models were also considered when they provided mechanistic insight relevant to autoimmune pathogenesis. All retrieved publications were curated manually to confirm that they (i) identified a bacterial antigen and (ii) provided evidence of its involvement in autoimmunity through a defined molecular mechanism. In addition to direct experimental validation, studies presenting biologically plausible indirect mechanistic evidence, such as molecular mimicry or immune activation pathways, were retained when supported by peer-reviewed literature. No conflicting evidence was identified among the selected studies, and multiple independent publications were used where available to support each curated antigen–mechanism relationship.

Manual curation produced a catalogue of 73 immunogenic antigens. For each antigen, details collected from the literature, such as the name, source organism, and reported mechanism of immune modulation, were recorded. Then the list of antigen names was provided to custom Perl 5.34 scripts that queried the UniProt REST API to download every associated reviewed and unreviewed protein entry for each antigen. These sequences constituted the core dataset for the downstream processing workflow and the database construction detailed in the following sections.

### Data processing

During download, basic metadata—including entry count, minimum and maximum sequence length—were computed and stored. Sequence headers were then normalized to the convention <AntigenID>_<counter>, providing unique, traceable identifiers for downstream analyses. Redundant entries were collapsed with MMseqs2 v15.6f452 at a 100% sequence-identity threshold, yielding a non-redundant representative set that still preserved closely related antigen variants for downstream analyses. The curated FASTA files, per-antigen statistics, and cluster membership tables were consolidated into a comma-separated values (CSV) archive that underpins the GUTAID web interface.

### Database implementation

**Figure 1:**
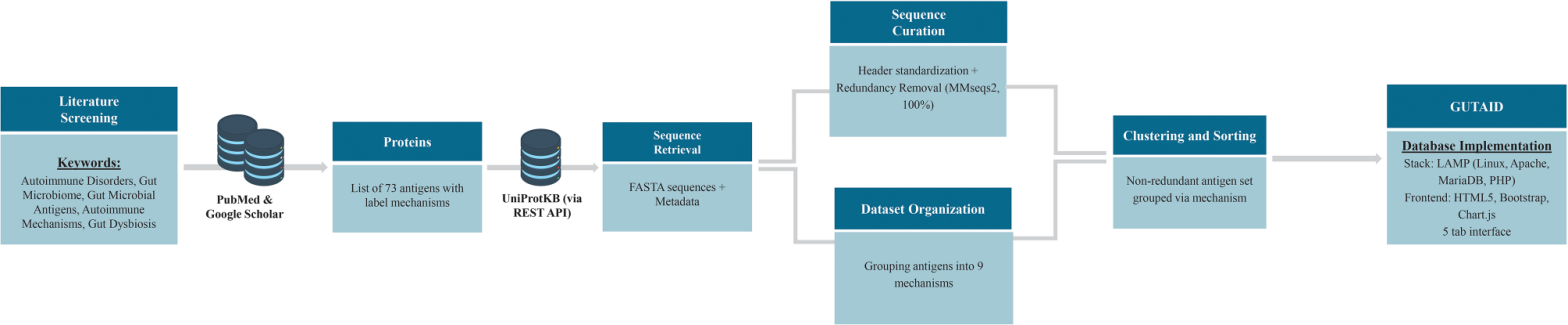
Framework for developing GUTAID.

The GUTAID web application was implemented on a conventional LAMP architecture (Linux, Apache, MySQL/MariaDB, PHP). Server-side functionality was developed in PHP 8.2 and deployed on a Linux host running Apache 2.4. Curated data were stored in a relational MariaDB 10.10.2 instance comprising two core tables, *mechanism* and *antigen*. Pages were rendered server-side with HTML5 and Bootstrap 3.0.2, while client-side interactivity used vanilla JavaScript and Chart.js for data visualization. Query performance was optimized with indexed fields and offset-based pagination (10 records per request), which adequately supported the current dataset without external caching or full-text search. Each antigen record was assigned a unique, auto-incremented identifier; all traffic was served over HTTPS. Administrative updates were executed via a secure CSV importer, and non-redundant FASTA archives were distributed from a dedicated/FASTA_files/directory. The codebase was maintained in a private Git repository and made available for academic research on request. The framework of the database construction is illustrated in Fig. 1 and Supplementary Fig. 1.

Results

## Database contents and usage

GUTAID hosts 73 experimentally verified bacterial antigens, each assigned to one or more of nine literature-derived autoimmune mechanisms. Table 1 presents the mechanism-wise distribution of these antigens and the corresponding sequence totals identified in this study. Every entry combines a non-redundant UniProt sequence cluster with mechanism tags and basic statistics, all accessible through a streamlined web interface. Users can browse, search, or download the full dataset via five dedicated tabs—Home, Downloads, Search, Team, and About.

**Table 1:** Mechanism-wise summary of antigens and their corresponding total sequences across non-mutually exclusive, literature-derived autoimmune mechanism categories.^a^

Major mechanism	Number of antigens	Percentage of total (73)	Total sequence
Molecular mimicry	34	46.58%	1 194 149
Superantigen activation	4	5.48%	3 652
Immune activation	25	34.25%	708 105
Epitope spreading	3	4.11%	4 052
Chronic infection	5	6.85%	782 480
Protein citrullination	3	4.11%	10 874
Immune modulation	11	15.07%	559 101
Inflammation induced response	15	20.55%	603 842
Immune system disruption and others	3	4.11%	287 807

a Sequence counts represent post-clustering totals at 100% sequence similarity

The specific functions offered by each of these pages are detailed in the sections that follow.

### Home

The Home page greets visitors with a brief mission banner—linking to the ‘About’ section for fuller context—followed by an icon grid of the nine key molecular mechanisms indexed in GUTAID. Selecting any mechanism opens a dedicated view where antigens associated with that pathway are listed in a paginated table displaying their names, mechanism descriptors, sequence-length range, and cluster size, alongside a one-click FASTA download for each entry. A pie chart on the Home page summarizes the overall mechanism distribution, highlighting molecular mimicry as the most frequent category.

### Downloads

The Downloads section provides direct access to the curated sequence sets. All nine molecular mechanisms are listed on the Downloads page; clicking any mechanism starts an instant download of a compressed FASTA file of all sequences associated with that particular pathway. A separate ‘Download All FASTA’ button packages the entire collection—covering every mechanism—into a single archive for users who need a complete export. This layout supports both focused and comprehensive data retrieval without requiring additional navigation.

### Search

The Search page offers a complete, tabular view of every antigen in GUTAID, displaying its mechanism label, sequence-length range, cluster size, and a direct FASTA download link. A case-insensitive query bar at the top of the search page supports keyword retrieval by antigen name, molecular mechanism, or a combination of both, instantly filtering the table to matching records. This layout enables rapid identification of any antigen in the database while preserving access to the same statistical details provided elsewhere in the database.

### Team

The Team page lists the Principal Investigator, Research Scientist, and Database Developer from the Metagenomics Discovery Lab, National University of Sciences & Technology. Institutional affiliation and contact e-mail are provided for each team member, promoting transparency and enabling direct communication with the individuals responsible for GUTAID’s conception, curation, and technical development.

### About

The About page outlines the scientific rationale for GUTAID, traces its origins in metagenomic research, and concisely explains the literature-driven and UniProt-based methodology used to curate gut microbial antigens. Collectively, these details furnish users with the context required to understand the breadth and scientific significance of the database.

**Figure 2:**
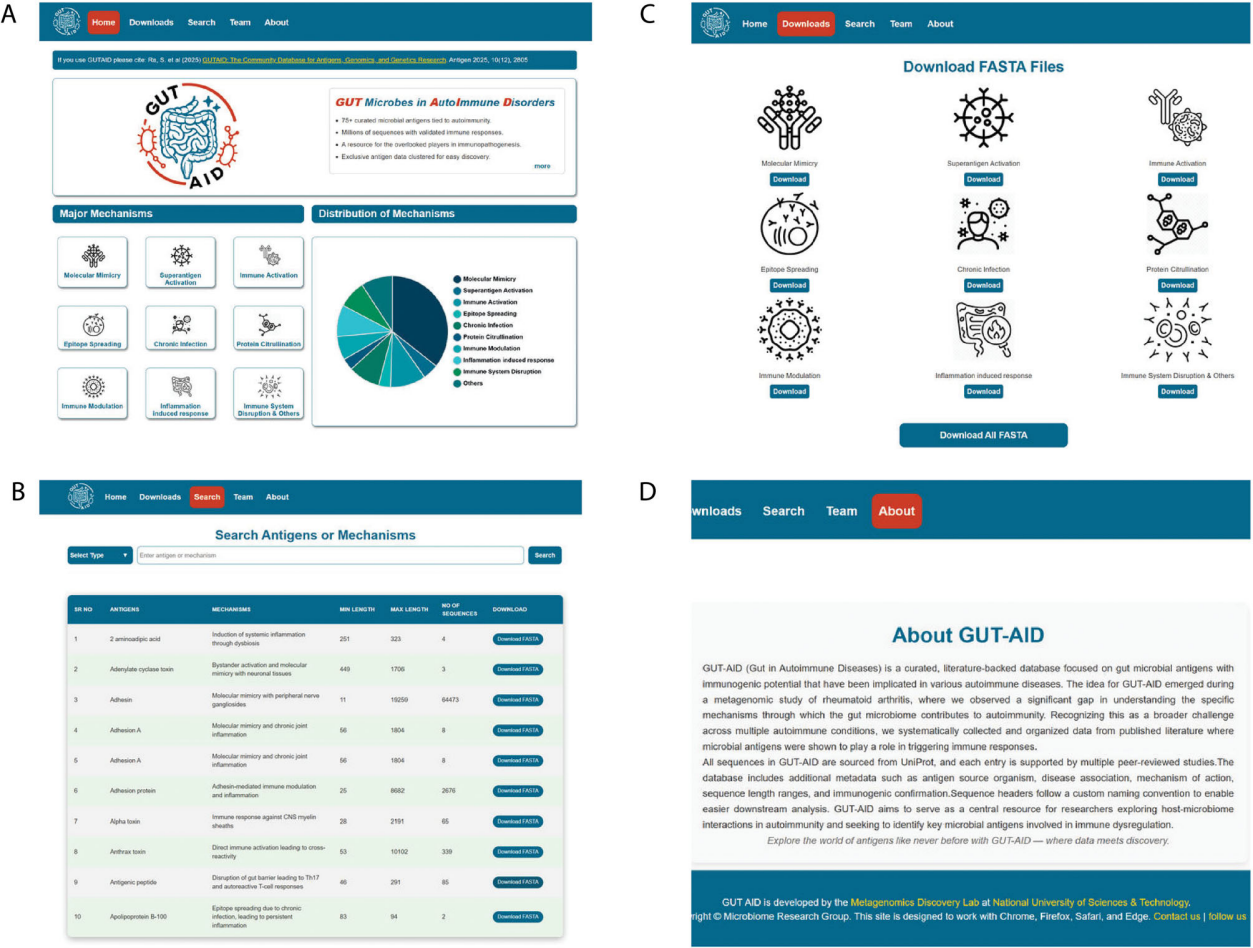
The graphical user interface of GUTAID. (A) The Home page of GUTAID. (B) The search page of GUTAID. The user can search for antigens via antigen name or mechanism or both. (C) The Download page of GUTAID allows the user to download antigen sequences by mechanism. (D) The About page of GUTAID.

The graphical user interface of GUTAID is shown in Fig. 2.

### An example of usage

To demonstrate the usage of GUTAID, we queried heat-shock protein 65 (Hsp65) from *Mycobacterium tuberculosis*, an antigen repeatedly implicated in autoimmune pathogenesis through molecular mimicry and cross-reactive anti-Hsp65 responses [28–30]. On the Search page, the user first selects Antigen as the query type and then enters any common synonym (‘HSP65’, ‘hsp65’, or ‘heat shock protein 65’). The case-insensitive filter instantly returns a single row summarizing the record: tagged mechanisms (molecular mimicry; immune response to bacterial heat-shock proteins), sequence-length span (63–541 aa), and a 401-member non-redundant cluster, together with a one-click Download FASTA link. This allows immediate retrieval of every curated Hsp65 sequence for downstream analysis, while the table itself provides a concise snapshot of the antigen’s immunological context.

The same interface supports pathway-based exploration. Switching the query type to mechanism and entering ‘molecular mimicry’ repopulates the table with all antigens assigned to that category, each accompanied by its own statistics and individual download button. For users who prefer a bulk export, the Downloads page offers a single ‘molecular mimicry’ tile that delivers a compressed FASTA archive encompassing every mimicry-associated antigen in GUTAID. Together, these two search options, single-antigen queries and mechanism-level downloads, show how GUTAID simplifies access to curated sequences for research on microbe-driven autoimmunity.

## Conclusion

GUTAID is the first specialized repository that unites literature-verified gut microbial antigens with explicit autoimmune-mechanism tags and non-redundant UniProt sequences. Its lightweight LAMP infrastructure and streamlined download options give researchers rapid, mechanism-aware access to 73 antigens spanning nine molecular mechanisms, providing an immediate resource for dissecting host–microbiome interactions in autoimmunity.

The current version of GUTAID should be considered the first release of an expanding reference resource. The present version reflects a deliberately conservative curation strategy based primarily on PubMed and Google Scholar literature and restricted to bacterial antigens with experimentally supported links to autoimmune disease. Accordingly, the current coverage may be influenced by literature availability and reporting bias, and fungal or viral antigens were not included in this initial release. All sequence sets are presently represented at a 100% sequence-identity clustering threshold, while future versions will also provide additional sequence collections at multiple clustering levels. Future work will expand both content and capacity. Scheduled literature searches and UniProt synchronizations will add newly reported antigens, while back-end upgrades—caching, full-text indexing, and a public MIT-licensed GitHub release—will improve query performance, scalability, and community involvement. These developments will ensure that GUTAID remains a robust, up-to-date platform for advancing microbe-mediated autoimmunity research.

## Supplementary Material

baag029_Supplemental_Files

## Data Availability

The publicly retrieved gene sequences and relevant details are available from the following URL: https://gutaid.mgdiscoverylab.com/. Supplementary material, including a complete list of bacterial antigens, their mechanism, and supporting references, and a list of bacteria reported in autoimmune diseases are shared as Supplementary Tables 1 and 2, respectively. This data has also been shared on Zenodo: https://doi.org/10.5281/zenodo.19661956
