# Supplementary material for: GUTAID: a curated database linking gut microbial antigens to autoimmune mechanisms
Source: Database (Oxford). 2026 Jun 3;2026:baag029. doi: 10.1093/database/baag029 (PMC13231167; doi:10.1093/database/baag029)
Supplement: baag029_Supplemental_Files [file baag029_supplemental_files.zip › Supplementary Figure 1.docx]

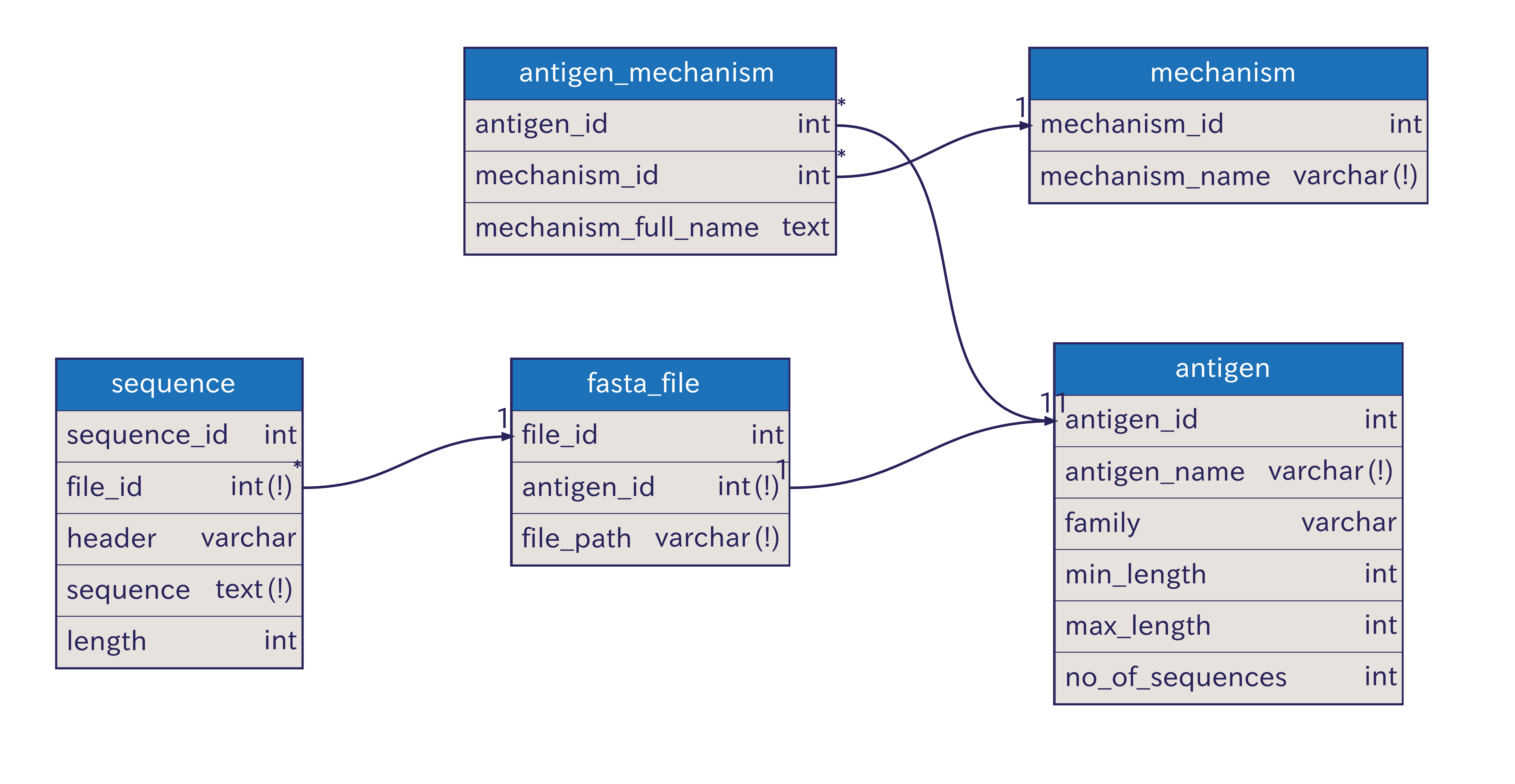


**Supplementary Figure 1: Entity–Relationship Diagram of the GUTAID Database.** The diagram shows the five core tables of GUTAID (*mechanism*, *antigen*, *antigen_mechanism*, *fasta_file*, *sequence*) and the relationships between them, illustrating how each microbial antigen is linked to one or more major immune mechanisms and to its curated protein sequences stored in a per-antigen FASTA file.
